# Mutation extraction tools can be combined for robust recognition of genetic variants in the literature

**DOI:** 10.12688/f1000research.3-18.v2

**Published:** 2014-06-10

**Authors:** Antonio Jimeno Yepes, Karin Verspoor

**Affiliations:** 1 National ICT Australia, Victoria Research Laboratory, Melbourne, Australia; 2 Department of Computing and Information Systems, The University of Melbourne, Melbourne, Australia

## Abstract

As the cost of genomic sequencing continues to fall, the amount of data being collected and studied for the purpose of understanding the genetic basis of disease is increasing dramatically. Much of the source information relevant to such efforts is available only from unstructured sources such as the scientific literature, and significant resources are expended in manually curating and structuring the information in the literature. As such, there have been a number of systems developed to target automatic extraction of mutations and other genetic variation from the literature using text mining tools. We have performed a broad survey of the existing publicly available tools for extraction of genetic variants from the scientific literature. We consider not just one tool but a number of different tools, individually and in combination, and apply the tools in two scenarios. First, they are compared in an intrinsic evaluation context, where the tools are tested for their ability to identify specific mentions of genetic variants in a corpus of manually annotated papers, the Variome corpus. Second, they are compared in an extrinsic evaluation context based on our previous study of text mining support for curation of the COSMIC and InSiGHT databases. Our results demonstrate that no single tool covers the full range of genetic variants mentioned in the literature. Rather, several tools have complementary coverage and can be used together effectively. In the intrinsic evaluation on the Variome corpus, the combined performance is above 0.95 in F-measure, while in the extrinsic evaluation the combined recall performance is above 0.71 for COSMIC and above 0.62 for InSiGHT, a substantial improvement over the performance of any individual tool. Based on the analysis of these results, we suggest several directions for the improvement of text mining tools for genetic variant extraction from the literature.

## Introduction

As the cost of genomic sequencing continues to fall, the amount of data being collected and studied for the purpose of understanding the genetic basis of disease is increasing dramatically. There are large-scale efforts to catalog the results of this research in structured databases, including the Online Mendelian Inheritance in Man (OMIM) database ^1^ and the Human Gene Mutation Database (HGMD) ^2^. Much of the source information relevant to such efforts is available only from unstructured sources such as the scientific literature, and significant resources are expended in manually curating and structuring the information in the literature. As such, there have been a number of systems developed to target automatic extraction of mutations and other genetic variation from the literature using text mining tools ^3– 9^, *inter alia*. Such tools have been shown to perform well, benefiting from a well-defined target vocabulary (nucleic and amino acids), the availability of reference sequences for position validation, and increasing adoption of standard nomenclature such as the Human Genome Variation Society (HGVS) format ^10^. The natural language descriptors of genetic variation are fairly consistent, and lend themselves well to automated processing.

In previous work ^11, 12^, we assessed one of these tools, the Extractor of Mutations (EMU) tool ^6^, for its ability to identify the genetic variant information that had been manually curated in the COSMIC ^13^ and the International Society for Gastro-intestinal Hereditary Tumours (InSiGHT) ^14^ databases from targeted literature sources. That work found very low recall for the text mining tool when considering the narrative content of publications alone, and identified processing of the supplementary material associated with publications as a critical component of an approach to automated genetic variant curation from the literature.

In this work, we perform a broad survey of the existing publicly available tools for extraction of genetic variants from the scientific literature. We consider not just one tool but a number of different tools, individually and in combination, and apply the tools in two scenarios. First, they are compared in an *intrinsic* evaluation context, where the tools are tested for their ability to identify specific mentions of genetic variants in a corpus of manually annotated papers, the Variome corpus ^15^. Unlike previous test corpora, this corpus was not designed exclusively for the purpose of testing mutation extraction tools and hence is a better test of real-world applicability than prior corpora. Second, they are compared in an *extrinsic* evaluation context based on our previous study with COSMIC and InSiGHT. Our results demonstrate that several of the tools have complementary coverage and can be used together effectively. This study suggests several directions for the improvement of text mining tools for genetic variant extraction from the literature.

## Background

Text mining of mutations in the scientific literature has been addressed by several tools, including MutationMiner ^3^, MarkerInfoFinder ^16^, EMU (Extractor of Mutations) ^6^, MutationFinder ^4^, tmVar ^9^, and SETH ^17^. A summary of previous work can be found in Naderi and Witte (2012) ^7^. These tools have been shown to achieve a performance over 0.90 in F1 measure, and in some cases almost perfect Precision/Recall, on intrinsic evaluations. There are also several corpora that are publicly available to support intrinsic evaluation of mutation extraction tools ^4, 6, 16, 18– 20^. On the other hand, cross-comparison of these tools has been limited. The most commonly used is the corpus provided with the MutationFinder tool, which covers protein variants. Some of these tools have also been used to reproduce the information curated in existing databases about genetic variants, allowing for extrinsic evaluation of the mutation extraction tools.

### Text mining tools for genetic variant extraction

In the following sections, we present the tools for genetic variant extraction that we have considered in our study. Each is a publicly available tool. The tools are introduced and their published results in intrinsic evaluation are presented. Results are presented in terms of precision (P), recall (R) and F1 measure (F).

### MutationFinder

MutationFinder (MF) was one of the earliest tools developed for extraction of mutations. This tool performs point mutation extraction based on a set of regular expressions ^4^. The coverage of this tool is thus limited compared to the other ones. A corpus, the MutationFinder corpus, was established to guide the construction of the patterns. The development data set is made up of 605 point mutation mentions in 305 abstracts selected randomly from primary citations in PDB. The evaluation data set is made up of 910 point mutation mentions in 508 abstracts annotated by two of the authors, not involved in the development of the system. Mean pairwise interannotator agreement, calculated on the fifty overlapping abstracts, was 94% ^4^. Performance of MF in the extracted mutations is P: 0.984 R: 0.817 F: 0.893.

*Technical Notes:* MutationFinder is implemented in Java. It is available either stand-alone or integrated into UIMA. It delivers the protein mutation with information about its position in text. It was easy to integrate in a Java program.

### Open Mutation Miner

Open Mutation Miner (OMM) is a tool ^7^ that extracts protein mutation mentions and maps them to their properties in the OMM impact ontology, which covers protein mutation types (insertion, deletion, point mutation), protein types and the impact of the mutation. The extraction of mutations component couples grammar rules with a normalization step, e.g. transformation into single-letter format. The system is combined with MutationFinder to identify variants in natural language. OMM has been developed under the GATE platform and it is available as a JAPE-based mutation tagging component. Mutation extraction was evaluated on a set of 11 full text articles, with an average performance of P: 0.99, R: 0.96 and F: 0.97 ^7^.

*Technical Notes:* OMM required installing GATE (General Architecture for Text Engineering, https://gate.ac.uk), which needs additional components that are required for mutation annotation. It delivers the protein mutation annotations, their position in text and information about the mutated and wild types. In addition, using OMM requires learning to work with GATE, which has a non-trivial learning curve.

### Extractor of Mutations

The Extractor of Mutations (EMU) tool ^6^ was designed to capture a broader range of mutations than other tools available when it was developed and hence is a better fit for the variants we might expect to find. It identifies protein and DNA point mutations, Single Nucleotide Polymorphism Database (dbSNP) identifiers ^21^ (RSIDs), and DNA insertions and deletions. In addition, it links the mutations to the proteins and genes that appear in text and performs sequence verifications using existing sequence databases to increase the precision of the annotations. EMU has been shown to have a performance of 0.92 F1 measure on an intrinsic evaluation ^6^, i.e., it has high recall and high accuracy.

*Technical Notes:* EMU is implemented in Perl, and thus could not easily be called from Java either. We built a wrapper around it to annotate the Variome corpus. For the extrinsic analysis, we produced files compliant with EMU’s file format and post-processed the output. Position of the mutation in text is not provided by the tool; it was done by matching the mapped string to produce the EMU annotations for Variome corpus.

### tmVar

tmVar ^9^ ( http://www.ncbi.nlm.nih.gov/CBBresearch/Lu/pub/tmVar) is a recently released mutation extraction tool based on a conditional random field model used with a special set of features, which has shown to perform better than MutationFinder on the MutationFinder corpus. tmVar aims to cover a wide range of sequence variants in both protein and gene levels in HGVS format. tmVar has been trained on 334 newly annotated citations, different from the MutationFinder corpus, and evaluated using the MutationFinder test set (P: 0.9880, R: 0.8962, F: 0.9398) and using their own data set made up of 134 manually annotated test citations (P: 0.9138, R: 0.9140, F: 0.9139) ^9^.

*Technical Notes:* tmVar is implemented in Perl. Thus we converted the documents to a text file format accepted by the tool and post-processed the output to identify the different components for our evaluation. A new version of tmVar allows using BioC format ^22^ for input/output. There was a problem in the input format produced for tmVar that required repetition of the Variome corpus experiments. Post-processing of the files produced by tmVar is required before integrating it with other software components.

### SNP Extraction Tool for Human Variations

SNP Extraction Tool for Human Variations (SETH) ( http://rockt.github.io/SETH) implements an Extended Backus–Naur Form (EBNF) grammar proposed by Laros *et al.* ^23^ to identify mentions of mutation that obey the HGVS nomenclature. Since mentions in text might not follow the HGVS nomenclature, SETH integrates MutationFinder to extend its coverage. SETH returns whether a mutation is a DNA or protein variant and the type of variant (e.g. *deletion*). Since we are already comparing the performance of MutationFinder, we have not run SETH with MutationFinder activated, so higher recall might be obtained when using the version that includes MutationFinder. This implies that SETH’s performance will be different if MutationFinder is activated.

SETH has been evaluated on several corpora, as reported on the SETH web site. Among other corpora, SETH evaluation has been performed on the MutationFinder test set (Precision 0.97 Recall 0.83 F: 0.89), the tmVar corpus (P: 0.94, R: 0.81, F: 0.87), the Thomas *et al.* corpus ^24^ (P: 0.95, R: 0.58, F: 0.72) and the Osiris corpus ^25^ (P: 0.98, R: 0.85, F: 0.91).

*Technical Notes:* SETH provides detailed information about the mutation, including a classification into several mutation types. SETH has been implemented under SCALA ( http://www.scala-lang.org), which we were unfamiliar with. We integrated it with Java building a jar file from the SETH distribution and called it from Java directly instead of using a SCALA program.

### Evaluation contexts for automated genetic variant extraction

***Intrinsic evaluation: annotated corpora.*** There are several text corpora that have been made available for the evaluation of mutation extraction tools. There are corpora that focus on protein mutations alone, on protein and DNA mutations or on normalizing the mentions to dbSNP identifiers.

Several corpora are available for the evaluation of protein mutation extraction tools. As presented above, the developers of MutationFinder ^4^ made available a data set ( http://mutationfinder.sourceforge.net) of 305 abstracts annotated with point mutations that was used for system development and 508 abstracts available for evaluation. The developers of OMM ^7^ ( http://www.semanticsoftware.info/open-mutation-miner) performed experiments on 11 full text articles annotated manually with protein mutations, although these documents are not publicly available for distribution, the manual annotations are available with the tool download. Other corpora exist annotated with protein residue information either manually annotated ^18, 19^ or prepared using automatic methods ^20^.

There are corpora available that contain both protein and DNA mutations. The EMU corpus ^6^ ( http://bioinf.umbc.edu/EMU/ftp) was developed for annotation of mutations related to prostate cancer. This data set was developed by querying MEDLINE for the medical subject heading (MeSH) *Mutation* and selecting citations relevant for prostate cancer based on MetaMap annotation. It contains 500 manually annotated abstracts with 95 mutations in 55 abstracts. The tmVar ^9^ ( http://www.ncbi.nlm.nih.gov/CBBresearch/Lu/pub/tmVar) system also comes with its own annotated set. The set comprises 500 abstracts manually annotated from which 334 were used for training tmVar while the remaining 166 were used for testing it. These citations were recovered from PubMed selecting English abtracts containing novel human mutations, targeting formulaic mentions. The Variome corpus ^15^ ( http://www.opennicta.com/home/health/variome) is an annotated biomedical textual resource pertaining to human genetic variation and its relation to diseases and other entity types. At present, the corpus comprises ten double annotated full text journal publications on inherited colorectal cancer, which were selected on the basis of their relevance to the genetics of the Lynch Syndrome to support the curation of the InSiGHT database.

There are two corpora to evaluate the normalization of extracted mutations to dbSNP identifiers. OSIRIS ^25^ ( https://sites.google.com/site/laurafurlongweb/databases-and-tools/corpora) was collected from MEDLINE covering English abstracts for human mutation, limited to 2004 and 2005 and focused on specific project criteria. The annotation is performed focused on evaluation of entity recognition and of disambiguation of variation entities to dbSNP identifiers. The final version of the corpus contains 105 abstracts available with 109 normalized variants and 155 unnormalized ones. The second corpus, developed by Thomas *et al.* ^24^ ( http://www.scai.fraunhofer.de/snp-normalization-corpus.html), consists of 296 MEDLINE abstracts annotated with 527 mutationdbSNP id pairs. The corpus was annotated initially with MutationFinder and then manually annotated for completion and normalized to dbSNP. Mutations without a valid dbSNP identifer were removed. Only the annotations are available, while the abstracts can be downloaded from PubMed using NCBI’s E-utilities ( http://www.ncbi.nlm.nih.gov/books/NBK25500).

Table 1 summarizes the results of the tools presented in the previous section as reported on available corpora. We can see that not many tools have been evaluated with the same corpus, other than the MutationFinder test set. The different tools evaluated on the MutationFinder corpus show that the performace in precision is generally very high across the tools with some differences in recall. However, the MutationFinder corpus covers only a limited set of protein mutations. In this work, we perform an intrinsic evaluation of the tools using the Variome corpus as the common reference set, producing a broader comparison of the different mutation extraction tools. We will introduce this corpus in detail in the Methods section.

**Table 1.** Mutation extraction systems evaluation on existing corpora. The results in precision, recall and F1 measure are obtained from already published studies or the tool website like SETH. A dash(-) is used to indicate that no results are available. The tools are MutationFinder (MF), OpenMutationMiner (OMM), Extractor of Mutations (EMU), tmVar and SNP Extraction Tool for Human Variations (SETH). The corpora used for evaluation are MutationFinder corpora (MF), OpenMutationMiner corpora (OMM), EMU Prostate Cancer set (PCa), the tmVar corpora, the OSIRIS corpora and the corpora made available by Thomas *et al.* ^24^ (Thomas).

		Corpus					
Tool	Performance measure	MF	OMM	PCa	tmVar	Osiris	Thomas
MF	P R F	0.98 0.82 0.89	- - -	- - -	- - -	- - -	- - -
OMM	P R F	- - -	0.99 0.96 0.97	- - -	- - -	- - -	- - -
EMU	P R F	0.99 0.81 0.89	- - -	0.92 0.92 0.92	- - -	- - -	- - -
tmVar	P R F	0.99 0.90 0.94	- - -	- - -	0.91 0.91 0.91	- - -	- - -
SETH	P R F	0.97 0.83 0.89	- - -	- - -	0.94 0.81 0.87	0.98 0.85 0.91	0.95 0.58 0.72

***Extrinsic evaluation: curated databases.*** In addition to intrinsic evaluation, there have been several efforts in trying to reproduce the information curated in mutation databases through information extraction from the literature. A broader summary of extrinsic evaluation is available in Jimeno and Verspoor (2014) ^11^.

There have been several efforts with varying success in recovering the curated information from variants databases. Krallinger *et al.* ^5^ extracteded mutations from literature for the kinase domain from abstracts and full text showing different levels of coverage of KinMutBase ^26^, the Swissprot Variant database ^27^, SAAPdb ^28^ and the COSMIC database ^29^, for which only 6% of the mutations were recovered. Schenck *et al.* ^30^ worked on a small set of articles curated in COSMIC that they annotated, recovering up to 30% of their annotated mutations. Caporaso *et al.* ^31^, Nagel *et al.* ^18^ and Verspoor *et al.* ^32^ tried to recover information about protein mutations and residues annotated in PDB protein records with limited coverage when using abstracts and with larger, but still very limited, coverage when using full text. On the pharmacogenomics side, Rance *et al.* ^33^ and Hakenberg *et al.* ^8^ tried recovering variants and related drugs to reproduce the data in PharmGKB with different coverage depending on the target genes.

In a previous study ^11^, we evaluated the ability of a mutation extraction tool to recover the curated mutations in the COSMIC and InSiGHT databases using the articles that were curated in these databases. We found, as in previous studies, that the recall considering the mutations extracted from the abstracts was very low. When considering the full text of these articles the recall increased but was still low. We found that many of the missing mutations were extracted by EMU from tables and supplementary material. In our current work, we have expanded this study by performing an intrinsic evaluation of several mutation extraction tools using the Variome corpus and performing a coverage evaluation of the tools when recovering the curated mutations from COSMIC and InSiGHT. Sample code and the files used for evaluation have been made available from a bitbucket repository: https://bitbucket.org/readbiomed/mutationtoolcomparison/.

## Methods

We have performed the evaluation and comparison of mutation extraction tools intrinsically, using the Variome corpus ^15^, developed in collaboration with the Human Variome Project ( http://www.humanvariomeproject.org), as a reference set. For the extrinsic study, we required a curated database that includes mutations and specific links to the literature (with PubMed identifiers [PMIDs] included for each mutation). We selected the COSMIC and InSiGHT databases for our investigation. These databases are used as reference sets; the information extracted from the corresponding scientific literature is compared directly to the information curated from those articles in the databases. We normalize extracted mutation mentions to Human Genome Variation Society (HGVS) format ^10^.

### The Variome corpus

The Variome corpus ^15^ ( http://www.opennicta.com/home/health/variome) is an annotated resource of biomedical texts pertaining to human genetic variation and its relation to diseases and other related entity types. At present, the corpus comprises ten double annotated full text journal publications on inherited colorectal cancer, which were selected on the basis of their relevance to the genetics of the Lynch Syndrome to support the curation of the InSiGHT database. The annotation schema covers thirteen relations, such as *gene-has-mutation*, *mutation-has-size* and *disease-related-to-bodypart*; and eleven entity types, such as genomic categories (e.g., *gene*, *mutation*), phenotypic categories (e.g., *disease*, *body-part*), categories related to the occurrence of mutations in a disease (e.g., *age*, *ethnicity*), and a *characteristic* category for the eventual addition of relevant information. Compared to other variation corpora, the Variome corpus not only annotates mutation mentions but also other entity types, providing a larger set of relevant entities. In addition, it contains annotations for relations between the entities, which provide a more exhaustive context for the training and evaluation of text mining tools supporting the curation of genetic variant databases.

The mutation entity type captures mentions of mutations which specify changes in the protein or DNA sequence as well as mutation terms which refer to general properties of a mutation (e.g. *somatic mutation*) or terms specifying a mutated gene (e.g. *APC+*). Current mutation extraction tools are only concerned with the first type, thus extracting mentions of protein or DNA changes. We have manually catalogued the annotated mutations and identified 118 mutation instances that are annotated in the corpus. From this set, 52 are DNA mutations and 66 are protein mutations.

### The mutation databases

In this work, we expand our previous study ^11^ and retain the original data sets for ease of comparison.

COSMIC ^13^ ( http://www.sanger.ac.uk/cosmic) contains comprehensive, curated, information on somatic mutations in human cancer. We used version v62 (from 29th of November 2012) available from COSMIC’s FTP site ( ftp://ftp.sanger.ac.uk/pub/CGP/cosmic/), including mutation information curated from 9,950 unique PubMed articles, as well as Cancer Genome Project (CGP) ( http://www.sanger.ac.uk/genetics/CGP) studies and international system screens (e.g. International Cancer Genome Consortium (ICGC) ( http://dcc.icgc.org/web)). We identified 7,898 publications associated to mutation information in this resource. cDNA and protein mutation information is already available in HGVS format. Genes are referenced by name and by HGNC (HUGO Gene Nomenclature Committee) identifier.

InSiGHT ^14^ maintains a database of genetic variants for both Lynch Syndrome and Familial Adenomatous Polyposis. The current database only has curated mutations for four genes: *MLH1*, *MSH2*, *MSH6* and *PMS2*. The original database was established in the 1990s with mutations reported by individual laboratories. Reports manually extracted from published literature currently comprise the majority of entries in the InSiGHT database (approximately 75%, according to the database curator), with the balance direct submissions from clinics.

We accessed the InSiGHT database on 02 January 2013 to establish our data set. The data includes variants with curated associations linked to 809 PubMed citations. The database contains information about the variants in the fields *Variant/DNA* and *Variant/Protein*. The amino acids in protein variants have been normalized to single letter amino acid abbreviation form.

There are 41 articles that have been curated both in COSMIC and InSiGHT databases. Unfortunately, none of the mutations in the overlapping articles has been curated by both databases because COSMIC is focused on somatic mutations, while InSiGHT is focused on germline mutations in only four genes.

### Corpus collection

An abstract for each PMID was retrieved from MEDLINE using NCBI’s E-utilities. Abstracts were downloaded in XML format and XML escaped characters were converted to their text characters (e.g., A–&gt;T becomes A–>T). In the case of the COSMIC database, 17 articles did not seem to be available when querying PubMed.

A small portion of PubMed is available as full text articles through the Open Access collection in PubMed Central (PMC-OA). From the 9,950 PMIDs available from the COSMIC set only 563 were available from PMC-OA. From the 809 citations for InSiGHT only 12 were available through the full text PMC-OA. This represents less than 10% of the overall set referenced by both databases.

### Collection of articles’ additional material

In addition to narrative text, we have used the mutation extration tools with further content linked to the papers, which includes the tables and supplementary material and is representative of the broader full text literature ^34^. We collected articles from COSMIC and InSiGHT that are available in the open access part of PubMed Central (PMC), since it already contains the tables in the XML of the article and there are pointers to the supplementary material. For the set of 13 articles in the InSiGHT database that could be found in PMC, InSiGHT contains 252 mutation triples. COSMIC associates 33,814 mutation triples to the 563 articles in PMC.

We extracted the tables and table captions from the full text PMC articles. The COSMIC database references 394 PMC articles with tables; 197 of these were identified as having mutations in the tables. From the InSiGHT database there are only 8 articles with tables, of which 4 contain mutations. In these articles, no mutations were found in the abstract or full text.

Supplementary material was also identified from links within the PMC articles and downloaded. The InSiGHT set contains a limited number of supplementary material files (in 1/12 articles), while COSMIC has a larger number linked to the papers (in 138/563 articles). In contrast to PMC articles, available in XML following a consistent DTD (Document Type Definition), supplementary material appears in a variety of file formats. The most frequent types of supplementary material in this corpus, shown in Table 2, are, in order of frequency: MS Word documents, MS Excel spreadsheet, PDF documents, TIFF images and MS Powerpoint documents. Text from the supplementary material was extracted with Apache Tika 1.3 ( http://tika.apache.org/1.3). No image processing was performed.

**Table 2.** Count of supplementary file types. Each row denotes one of file type. For each type, for COSMIC the number of files and the number of articles, denoted by PMIDs, is shown. In the case of the InSiGHT database, there is only one supplementary file in MS Word format linked to one article.

Set	COSMIC		InSiGHT
	Files	PMIDs	
MS Word MS Excel PDF documents MS Powerpoint CSV files Images	176 111 82 34 1 101	87 57 70 17 1 36	1 0 0 0 0 0
Total	505	138	1

During the extraction of tables and supplementary material, we realized that some PMC articles do not contain the full text in XML format but a link to a PDF version of it. From the InSiGHT collection, 4 papers out of the 13 contained only the abstract with a link to the full text in PDF format. In the COSMIC collection, the proportion was 76 papers out of 563. The PDF version for these papers has been downloaded from the European PMC mirror ( http://europepmc.org), which offers a straightforward link to download the PDF files.

### Mutation identification in text

We have considered several state of the art mutation extraction tools in this study, as introduced in the *Text mining tools for genetic variant extraction* section above. We normalized the mutation mentions identified by these tools to follow the HGVS nomenclature, to be comparable to the information in the COSMIC and InSiGHT databases. This normalization required considering the specificities of each tool, thus a normalization program was prepared for each tool. Missense mutations, mutations in the DNA that result in a protein change, are normalized to amino acid (wild type), position, amino acid (mutated), using single letter amino acid abbreviations. Thus, a mutation identified with wild type amino acid *Ala*, position *140* and mutated amino acid *Thr* is converted to *A140T*. DNA mutations identified by any tool are normalized to the format “c.[position][wild type nucleotide]>[mutated nucleotide]”. In the case of insertion and deletions, given position ranges, hyphens are replaced by the underscore character (e.g. *c.597-598delGA* to *c.597_598delGA*).

We ignored EMU’s *Genome* category since genome variants do not appear in COSMIC or InSiGHT. We did not filter out mutation mentions based on sequence validation, so we consider all the extracted mutations from EMU. When EMU identifies a dbSNP identifier, the dbSNP API is queried to obtain further details about the mutations, identifying all available candidates for DNA and protein mutations associated with each ID. There were some mentions in which the position of the DNA or protein substitution mutation was provided as exon/intron number or a codon position. The codon positions were converted to the three candidate nucleotide positions. Exon and intron mentions were removed since no precise position could be derived.

### Gene normalization and linkage to the mutations

In the curated databases, the mutations are linked to the genes or proteins where they happen. In addition to the extraction of mutations, we have annotated and normalized the genes in the documents based on a dictionary developed from the NCBI Gene database, using only the human genes. We followed the procedure in Jimeno Yepes *et al.* (2013) ^35^ and removed duplicates and filtered out certain misleading or ambiguous gene names, such as those ending with disease, syndrome, or susceptibility, and removed terms from a standard stopword list. Based on observations from previous work ^12^, we have added the following variations to the genes related to the InSiGHT database. These terms are variations of the original gene term but prefixing the letter h to indicate that it is a human gene. Thus we have added *hMSH2* for *MSH2*, *hMSH6* for *MSH6* and *hPMS2* for *PMS2*.

We used our own dictionary because this has shown to be effective ^35, 36^ and human genes are not as ambiguous compared to other species. Since our objective is to investigate the coverage of current approaches when dealing with the curation of existing databases, we can have more control on the false positives and false negatives. In addition, we are considering resources in addition to MEDLINE citations and full text documents, for which current methods based on machine learning approaches do not need to perform as expected. We have used ConceptMapper ^37^ ( http://uima.apache.org/d/uima-addons-current/ConceptMapper/ConceptMapperAnnotatorUserGuide.html) as the dictionary tagger tool reusing the configuration prepared for the BioCreative 2013 CTD track ^36^, which does not make case distinction, tokens have to be matched in the same order and only the longest match is considered and tokens must be adjacent to each other. The identified genes are related to the mutations based on document co-occurrences.

## Results

We have performed two types of evaluation. An intrinsic evaluation of the variant extraction methods on an annotated corpus developed for the purpose of variant curation and an extrinsic evaluation based on the ability of the methods to recover the mutations from the articles.

### Mutation extraction on the Variome corpus

Intrinsic evaluation of the mutation extraction tools is shown in Table 3 in terms of precision, recall and F1 score. The results are estimated based on two matching schemas: exact matching, in which the annotated entities must match exactly the span of the entities in the reference set, and partial matching, in which the annotated entities may have any overlap with the entities in the reference set. The partial matching relaxes annotation boundaries, so entities with differences like DNA or protein variant prefixes *c.* and *p.* respectively are not considered as errors.

**Table 3.** Variant extraction results on the Variome corpus. Results for exact and partial matching are present. Each row shows the performance of each method in terms of true positives (TP), false negatives (FN), false positives (FP), Precision, Recall and F1 measure (F1). The tools are Extractor of Mutations (EMU), OpenMutationMiner (OMM), MutationFinder (MF), tmVar and SNP Extraction Tool for Human Variations (SETH) and their combination (either selecting the longest span Combined_longest or the shortest span Combined_shortest).

Exact	TP	FN	FP	Precision	Recall	F1	Char Overlap (%)
EMU OMM MF tmVar SETH Combined_shortest Combined_longest	66 7 7 81 81 34 78	52 111 111 37 37 84 40	25 56 10 26 10 76 32	0.7253 0.1111 0.4118 0.7570 0.8901 0.3091 0.7091	0.5593 0.0593 0.0593 0.6864 0.6864 0.2881 0.661	0.6316 0.0773 0.1037 0.7200 0.7751 0.2982 0.6842	100.00 100.00 100.00 100.00 100.00 100.00 100.00
Partial	TP	FN	FP	Precision	Recall	F1	Char Overlap (%)
EMU OMM MF tmVar SETH Combined_shortest Combined_longest	90 64 16 107 90 110 110	28 54 102 11 28 8 8	3 1 1 3 1 3 3	0.9677 0.9846 0.9412 0.9727 0.9890 0.9735 0.9735	0.7627 0.5424 0.1356 0.9068 0.7627 0.9322 0.9322	0.8531 0.6995 0.2370 0.9386 0.8612 0.9524 0.9524	82.08 70.01 52.91 81.11 80.99 82.03 83.03

Considering the partial matching, the precision of each tool is over 90%, which is in agreement with previously reported work on different corpora. On the other hand, the tools show different recall values. EMU, SETH and tmVar show the best performance, with SETH showing better results than EMU and tmVar in exact matching and tmVar showing better results in partial matching. MutationFinder has a significantly lower coverage due to its focus on point mutations. OMM displays better partial matching performance compared to MutationFinder, even though both systems only extract protein mutations. tmVar has lower exact matching performance than expected based on its previously reported performance. This might be due to the fact that tmVar was trained on abstracts, while the Variome corpus consists of full text articles. If we combine the annotations of the tools by simple merge, either selecting the largest or shortest annotation in the same span of text, when evaluating the results based on partial matching, the performance is higher than any single system (precision = 0.9735, recall = 0.9322 and F1 = 0.9524), in particular due to an increase in recall. When using exact matching, we find that selecting the longest match is better compared to the shortest one. This is because some systems annotated mutations without the prefix “p.”, which was annotated in the Variome corpus, e.g. *p.Lys618Ala*. This difference can have a substantial impact on recall in the partial matching scenario.

The Variome corpus contains not only abstracts but also full text. These results show that mutation extraction tools that were developed based on MEDLINE abstracts, once applied to narrative literature still have high precision, and that their combination provides a high precision and recall solution.

Since Open Mutation Miner and MutationFinder explicitly only deal with protein mutations, we have divided the results into DNA and protein mutation subsets and estimated recall for each subset, shown in Table 4 and Table 5 respectively. Unsurprisingly, OMM and MutationFinder did not recover any DNA mutation. The result on protein mutations show that MutationFinder has a very low performance, due to its coverage of only point mutations. Open Mutation Miner has a high recall, over 96%, as well as high precision as previously reported. tmVar has quite a high recall in the protein mutation set compared to the DNA mutation set. SETH has an overall higher performance but in this case, its recall is below EMU for protein mutation. This is because many mutations in text do not exactly follow the HGVS nomenclature. Considering DNA mutations, we find that except for SETH and tmVar, the performance of the other tools is lower. This is explained because there are specific types of DNA variants, e.g. deletions, that are not as well covered by the other tools as by SETH or tmVar. tmVar performance for DNA mutations is higher than any other system when partial matching is used and below SETH when exact matching is considered. As can be seen in Table 5, tmVar has much stronger coverage of protein mutations as compared to DNA mutations.

**Table 4.** DNA variant extraction recall result on the Variome corpus. Results for exact and partial matching are present. Each row shows the performance of each method in terms of true positives (TP), false negatives (FN) and Recall. The tools are Extractor of Mutations (EMU), OpenMutationMiner (OMM), MutationFinder (MF), tmVar and SNP Extraction Tool for Human Variations (SETH).

Exact	TP	FN	Recall	Char Overlap (%)
EMU OMM MF tmVar SETH	20 0 0 28 33	32 52 52 24 19	0.3846 0.0000 0.0000 0.5385 0.6346	100.00 100.00 100.00 100.00 100.00
Partial	TP	FN	Recall	Char Overlap (%)
EMU OMM MF tmVar SETH	33 0 0 43 40	17 52 52 9 12	0.6600 0.0000 0.0000 0.8269 0.7692	68.60 0.00 0.00 77.83 78.17

**Table 5.** Protein extraction recall result on the Variome corpus. Results for exact and partial matching are present. Each row shows the performance of each method in terms of true positives (TP), false negatives (FN) and Recall. The tools are Extractor of Mutations (EMU), OpenMutationMiner (OMM), MutationFinder (MF), tmVar and SNP Extraction Tool for Human Variations (SETH).

Exact	TP	FN	Recall	Char Overlap (%)
EMU OMM MF tmVar SETH	46 7 7 53 48	20 59 59 13 18	0.697 0.1061 0.1061 0.8030 0.7273	100.00 100.00 100.00 100.00 100.00
Partial	TP	FN	Recall	Char Overlap (%)
EMU OMM MF tmVar SETH	55 64 16 64 50	11 2 50 2 16	0.8333 0.9697 0.2424 0.9697 0.7576	95.12 70.01 52.91 84.18 83.72

Table 6 and Table 7 contain the frequency of false positives and false negatives made by each tool, grouped by type of genetic variation. Types of genetic variation were manually annotated. There are 8 DNA deletions, 2 DNA insertions/deletions, 42 DNA substitutions and 66 protein substitutions. In addition to a substitution that EMU identified incorrectly, all two other false positives are annotations that should be corrected in the Variome corpus. Specifically a protein substitution ( *V600E*) and a DNA deletion ( *c.2700_2701delTC*). EMU false negatives include deletions *c.3927_3931del AAAGA*, substitutions such as *c.1852_1853AA>GC* and mentions surrounded by parentheses. Considering the false negatives, MutationFinder failed at extracting mutations with three-letter amino acids (e.g., *p.Pro622Thr*) or single-letter amino acids without the *p.* prefix as *M23A*. OMM has only two false negatives that are protein mutations that appear within parentheses in text. SETH fails with expressions such as *C1668 C > T*, that should be *c.1668C > T*. All DNA mutation extraction tools fail with some expressions like *codon 41: A←G*, which might require additional regular expressions.

**Table 6.** DNA variant extraction error types and their frequency are presented for each system. Only the tools that perform DNA variant extraction are considered. The tools are Extractor of Mutations (EMU), tmVar and SNP Extraction Tool for Human Variations (SETH).

FP	Variant type	Count
EMU tmVar SETH	DELETION SUBSTITUTION DELETION SUBSTITUTION DELETION	1 1 1 1 1
FN	Variant type	Count
EMU tmVar SETH	SUBSTITUTION DELETION SUBSTITUTION SUBSTITUTION DELETION	12 5 9 10 2

**Table 7.** Protein variant extraction error types and their frequency are presented for each system. The tools are Extractor of Mutations (EMU), OpenMutationMiner (OMM), MutationFinder (MF), tmVar and SNP Extraction Tool for Human Variations (SETH).

FP	Variant type	Count
EMU OMM MF tmVar SETH	SUBSTITUTION SUBSTITUTION SUBSTITUTION SUBSTITUTION -	1 1 1 1 0
FN	Variant type	Count
EMU OMM MF tmVar SETH	SUBSTITUTION SUBSTITUTION SUBSTITUTION SUBSTITUTION SUBSTITUTION	11 2 50 2 16

### Mutation extraction for genetic variation curation

Results on the mutation extraction are available in Table 8 for the COSMIC database and in Table 9 for the InSiGHT database. The tables show results for different representations of the articles and different mutation extraction tools used. For each representation group, there is an additional row showing the result of combining the mutations extracted by each method. Each row shows the total number of mutations in the reference set, the number of mutations matched and the recall, which is just the proportion of the matched mutations with respect to the mutations in the reference set. Matching requires matching the complete triple {PMID, gene, mutation}.

**Table 8.** COSMIC variant extraction coverage result. The table shows the number of variants in the reference set (Total), the number of matched variants by the mutation extraction tool (Matched), the proportion of matched variants (Recall), the number of variants matched when the gene is not considered (M NG) and the proportion of matched variants when the gene is not considered (Rec NG). The data sets considered are MEDLINE abstracts (medline), Open Access PMC articles (pmc.ft), PDF articles when no Open Access PMC articles are available (pdf), PDF representation for all the articles (pdf.all), tables available from the Open Access PMC Articles’ XML (table), supplementary material (sup) and the combination from all the sources (all). The tools are Extractor of Mutations (EMU), OpenMutationMiner (OMM), MutationFinder (MF), tmVar and SNP Extraction Tool for Human Variations (SETH). The row with tool value as All indicates the result when the variants extracted by all the tools are merged.

Data set	Tool	Total	Matched	Recall	M NG	Rec NG
medline medline medline medline medline	EMU OMM MF SETH tmVar	33814 33814 33814 33814 33814	146 140 126 25 139	0.0043 0.0041 0.0037 0.0007 0.0041	157 147 137 26 145	0.0046 0.0043 0.0041 0.0008 0.0043
medline	All	33814	156	0.0046	169	0.0050
pmc.ft pmc.ft pmc.ft pmc.ft pmc.ft	EMU OMM MF SETH tmVar	33814 33814 33814 33814 33814	726 697 632 141 655	0.0215 0.0206 0.0187 0.0042 0.0194	758 726 658 148 682	0.0224 0.0215 0.0195 0.0044 0.0202
pmc.ft	All	33814	814	0.0241	853	0.0252
pdf pdf pdf pdf pdf	EMU OMM MF SETH tmVar	33814 33814 33814 33814 33814	34 1 5 6 4	0.0010 0.0000 0.0001 0.0002 0.0001	47 1 6 6 5	0.0014 0.0000 0.0002 0.0002 0.0001
pdf	All	33814	34	0.0010	47	0.0014
pdf.all pdf.all pdf.all pdf.all pdf.all	EMU OMM MF SETH tmVar	33814 33814 33814 33814 33814	1094 1132 989 246 1049	0.0324 0.0335 0.0292 0.0073 0.0310	1114 1137 996 247 1060	0.0329 0.0336 0.0295 0.0073 0.0313
pdf.all	All	33814	1304	0.0386	1327	0.0392
table table table table table	EMU OMM MF SETH tmVar	33814 33814 33814 33814 33814	580 597 462 179 176	0.0172 0.0177 0.0137 0.0053 0.0052	681 699 564 207 233	0.0201 0.0207 0.0167 0.0061 0.0069
table	All	33814	694	0.0205	831	0.0246
sup sup sup sup sup	EMU OMM MF SETH tmVar	33814 33814 33814 33814 33814	19177 20054 1286 21052 7763	0.5671 0.5931 0.0380 0.6226 0.2296	19217 20116 1308 21089 7782	0.5683 0.5949 0.0387 0.6237 0.2301
sup	All	33814	22756	0.6730	22829	0.6751
all all all all all	EMU OMM MF SETH tmVar	33814 33814 33814 33814 33814	20203 20960 2087 21335 8724	0.5975 0.6199 0.0617 0.6310 0.2580	20284 21040 2133 21379 8762	0.5999 0.6222 0.0631 0.6323 0.2591
all	All	33814	23859	0.7056	23969	0.7088

**Table 9.** InSiGHT variant extraction coverage result. The table shows the number of variants in the reference set (Total), the number of matched variants by the mutation extraction tool (Matched) and the proportion of matched variants (Recall). When relaxing the gene matching (M NG), the results do not change, thus this data is not shown. The data sets considered are MEDLINE abstracts (medline), Open Access PMC articles (pmc.ft), PDF articles when no Open Access PMC articles are available (pdf), PDF representation for all the articles (pdf.all), tables available from the Open Access PMC Articles’ XML (table), supplementary material (sup) and the combination from all the sources (all). The tools are Extractor of Mutations (EMU), OpenMutationMiner (OMM), MutationFinder (MF), tmVar and SNP Extraction Tool for Human Variations (SETH). The row with tool value as All indicates the result when the variants extracted by all the tools are merged.

Data set	Tool	Total	Matched	Recall
medline medline medline medline medline	EMU OMM MF SETH tmVar	252 252 252 252 252	1 4 0 1 4	0.0040 0.0159 0.0000 0.0040 0.0159
medline	All	252	5	0.0198
pmc.ft pmc.ft pmc.ft pmc.ft pmc.ft	EMU OMM MF SETH tmVar	252 252 252 252 252	23 5 1 3 22	0.0913 0.0198 0.0040 0.0119 0.0873
pmc.ft	All	252	26	0.1032
pdf pdf pdf pdf pdf	EMU OMM MF SETH tmVar	252 252 252 252 252	7 7 7 0 7	0.0278 0.0278 0.0278 0.0000 0.0278
pdf	All	252	8	0.0317
pdf.all pdf.all pdf.all pdf.all pdf.all	EMU OMM MF SETH tmVar	252 252 252 252 252	41 43 13 33 64	0.1627 0.1706 0.0516 0.1310 0.2540
pdf.all	All	252	85	0.3373
table table table table table	EMU OMM MF SETH tmVar	252 252 252 252 252	39 31 1 36 30	0.1548 0.1230 0.0040 0.1429 0.1190
table	All	252	74	0.2937
sup sup sup sup sup	EMU OMM MF SETH tmVar	252 252 252 252 252	88 0 0 0 92	0.3492 0.0000 0.0000 0.0000 0.3651
sup	All	252	92	0.3651
all all all all all	EMU OMM MF SETH tmVar	252 252 252 252 252	127 43 13 37 131	0.5040 0.1706 0.0516 0.1468 0.5198
all	All	252	157	0.6230

For the COSMIC database, most of the mutations are found in the supplementary material, while for InSiGHT we find that most of the mutations are spread between tables and supplementary material. Low recall is obtained from the mutations extracted from the articles’ abstracts and full text representations. This is in accordance with previously published work ^11, 12^, in which a similar effect is observed.

We explored the effect of combining the tested tools together, since it is apparent several have complementary scope. The combination was implemented simply by merging the results of the systems. The combination of all systems improves the previously published text mining coverage. Coverage increases from 45.63% recall to 62.30% in the case of the InSiGHT database and from 62.30% recall to 70.56% in the case of the COSMIC database.

The coverage of the COSMIC database is larger than the InSiGHT database. In InSiGHT, a large proportion of the information is found in tables in expressions that involve an intron position, i.e. *IVS17(+5)G>C*, which are not properly identified by the mutation extraction systems.

In addition to these results, we have relaxed the gene matching requirements, so only the PMID and mutation are required to match. In the result tables, these results are shown in the fields “ *M NG*” (NG=No Gene) for the number of matched mutations and the “ *Rec NG*” for the proportion of mutations covered from the reference set. We find that there is just a small increase in the case of the COSMIC database. There is no difference for the InSiGHT database and hence this additional data is not shown for the InSiGHT database. The InSiGHT database focuses on only four genes and the dictionary seems to cover all their possible gene name variations.

Considering the tools individually, MutationFinder has low coverage of the curated mutations. This result follows the observations from the intrinsic evaluation performed on the Variome corpus. OMM is focused on protein mutations, thus it also suffers from lower recall. Given its excellent performance in intrinsic protein variant extraction, this suggests that when its performance is low compared to other methods it means that DNA variants are more common, for instance in the supplementary material in the InSiGHT database. The recall of SETH in some cases is lower as compared to other methods. EMU provides a more robust coverage of mutations overall. However, the combination of different methods shows an increase compared to previous published work based only on this tool. This is partially explained by the coverage provided by OMM of protein mutations but also by the performance of SETH in the extraction of more complex deletions from the InSiGHT database.

During the analysis of the results, we realized that in a small number of cases PMC makes reference to the tables of the article without including their content. In the InSiGHT database this happens only with the PMID:12373605. To mitigate this problem we downloaded all the PDF files for all the PMC documents and converted them to plain text. This is given as *pdf.all* in the result tables. We find that this set contains more mutations than the full text or the table sets, because full text and tables are contained in the PDF of the articles.

There are articles for which no mutation extraction tool could recover any mutation. We have performed an analysis of the coverage of the mutation extraction tools for the articles in which at least one mutation can be identified by any mutation extraction tool and at least one mutation is in the reference set, referred to as the common set. The results on the common set are available from Table 10 and Table 11. Generally the coverage of the common set is higher. This difference is most dramatic when considering the citations for which mutations can be identified in the abstract, with 23% and 29% recall in COSMIC and InSiGHT respectively.

**Table 10.** COSMIC variant extraction coverage result when only articles with variants in the reference set as well as at least one result identified by the information extraction tool are considered. The table shows the number of common articles (PMIDs), the number of variants in the reference set (Total), the number of matched variants by the mutation extraction tool (Matched), the proportion of matched variants (Recall), the number of variants matched when the gene is not considered (M NG) and the proportion of matched variants when the gene is not considered (Rec NG). The data sets considered are MEDLINE abstracts (medline), Open Access PMC articles (pmc.ft), PDF articles when no Open Access PMC articles are available (pdf), PDF representation for all the articles (pdf.all), tables available from the Open Access PMC Articles’ XML (table), supplementary material (sup) and the combination from all the sources (all). The tools are Extractor of Mutations (EMU), OpenMutationMiner (OMM), MutationFinder (MF), tmVar and SNP Extraction Tool for Human Variations (SETH). The row with tool value as All indicates the result when the variants extracted by all the tools are merged.

Data set	Tool	PMIDs	Total	Matched	Recall	M NG	Rec NG
medline medline medline medline medline	EMU OMM MF SETH tmVar	128 109 104 16 114	627 483 498 80 515	146 140 126 25 139	0.2329 0.2899 0.2530 0.3125 0.2699	157 147 137 26 145	0.2504 0.3043 0.2751 0.3250 0.2816
medline	All	137	676	156	0.2308	169	0.2500
pmc.ft pmc.ft pmc.ft pmc.ft pmc.ft	EMU OMM MF SETH tmVar	339 299 282 67 308	31742 31405 30415 1425 31391	726 697 632 141 655	0.0229 0.0222 0.0208 0.0989 0.0209	758 726 658 148 682	0.0239 0.0231 0.0216 0.1039 0.0217
pmc.ft	All	351	31848	814	0.0256	853	0.0268
pdf pdf pdf pdf pdf	EMU OMM MF SETH tmVar	61 8 16 2 32	474 64 131 10 294	34 1 5 6 4	0.0717 0.0156 0.0382 0.6000 0.0136	47 1 6 6 5	0.0992 0.0156 0.0458 0.6000 0.0170
pdf	all	64	505	34	0.0673	47	0.0931
pdf.all pdf.all pdf.all pdf.all pdf.all	EMU OMM MF SETH tmVar	439 341 330 83 379	33134 32428 31774 1555 32745	1094 1132 989 246 1049	0.0330 0.0349 0.0311 0.1582 0.0320	1114 1137 996 247 1060	0.0336 0.0351 0.0313 0.1588 0.0324
pdf.all	All	446	33295	1304	0.0392	1327	0.0399
table table table table table	EMU OMM MF SETH tmVar	197 166 146 38 90	1946 1765 1505 509 1128	580 597 462 179 176	0.2980 0.3382 0.3070 0.3517 0.1560	681 699 564 207 233	0.3499 0.3960 0.3748 0.4067 0.2066
table	All	211	2019	694	0.3437	831	0.4116
sup sup sup sup sup	EMU OMM MF SETH tmVar	77 86 76 37 73	27888 31156 30897 28088 30285	19177 20054 1286 21052 7763	0.6876 0.6437 0.0416 0.7495 0.2563	19217 20116 1308 21089 7782	0.6891 0.6457 0.0423 0.7508 0.2570
sup	All	106	31564	22756	0.7209	22829	0.7233
all all all all all	EMU OMM MF SETH tmVar	450 353 344 109 388	33409 32887 32623 29047 33008	20203 20960 2087 21335 8724	0.6047 0.6373 0.0640 0.7345 0.2643	20284 21040 2133 21379 8762	0.6071 0.6398 0.0654 0.7360 0.2655
all	All	458	33676	23859	0.7085	23969	0.7118

**Table 11.** InSiGHT variant extraction coverage result when only articles with variants in the reference set as well as at least one result identified by the information extraction tool are considered. The table shows the number of common articles (PMIDs), the number of variants in the reference set (Total), the number of matched variants by the mutation extraction tool (Matched) and the proportion of matched variants (Recall). When relaxing the gene matching (M NG), the results do not change, thus this data is not shown. The data sets considered are MEDLINE abstracts (medline), Open Access PMC articles (pmc.ft), PDF articles when no Open Access PMC articles are available (pdf), PDF representation for all the articles (pdf.all), tables available from the Open Access PMC Articles’ XML (table), supplementary material (sup) and the combination from all the sources (all). The tools are Extractor of Mutations (EMU), OpenMutationMiner (OMM), MutationFinder (MF), tmVar and SNP Extraction Tool for Human Variations (SETH). The row with tool value as All indicates the result when the variants extracted by all the tools are merged.

Data set	Tool	PMIDs	Total	Matched	Recall
medline medline medline medline medline	EMU OMM MF SETH tmVar	2 2 0 1 2	2 16 0 1 16	1 4 0 1 4	0.5000 0.2500 0.0000 1.0000 0.2500
medline	All	3	17	5	0.2941
pmc.ft pmc.ft pmc.ft pmc.ft pmc.ft	EMU OMM MF SETH tmVar	8 4 2 2 6	179 148 132 56 149	23 5 1 3 22	0.1285 0.0338 0.0076 0.0536 0.1477
pmc.ft	All	9	234	26	0.1111
pdf pdf pdf pdf pdf	EMU OMM MF SETH tmVar	3 2 2 0 3	18 14 14 0 18	7 7 7 0 7	0.3889 0.5000 0.5000 0.0000 0.3889
pdf	All	3	18	8	0.4444
pdf.all pdf.all pdf.all pdf.all pdf.all	EMU OMM MF SETH tmVar	11 8 6 2 11	251 241 185 56 251	41 43 13 33 64	0.1633 0.1784 0.0703 0.5893 0.2550
pdf.all	All	11	251	85	0.3386
table table table table table	EMU OMM MF SETH tmVar	4 4 3 1 2	197 197 142 55 118	39 31 1 36 30	0.1980 0.1574 0.0070 0.6545 0.2542
table	All	4	197	74	0.3756
sup sup sup sup sup	EMU OMM MF SETH tmVar	1 1 1 0 1	103 103 103 0 103	88 0 0 0 92	0.8544 0.0000 0.0000 0.0000 0.8932
sup	All	1	103	92	0.8932
all all all all all	EMU OMM MF SETH tmVar	12 8 6 2 11	252 241 185 56 251	127 43 13 37 131	0.5040 0.1784 0.0703 0.6607 0.5219
all	All	12	252	157	0.6230

When considering tables, the coverage of COSMIC seems to increase only slightly compared to the InSiGHT database. This might mean that many of the mutations are in tables in the InSiGHT database, while this is not the case for the COSMIC database.

Table 12 and Table 13 show the overlap of the mutations extracted by each tool using all the data sources from the articles. The results in the tables show the complementarity of the mutation extraction tools. MutationFinder has the lowest overlap with the mutations extracted by other systems, while EMU has the best coverage. The overlap of MutationFinder and OpenMutationMiner with other tools is lower in the InSiGHT database, which might indicate that there are proportionately more DNA mutations in this set compared to COSMIC.

**Table 12.** Overlap of mutations extracted by each tool on the COSMIC database from all article data sources. The tools are Extractor of Mutations (EMU), OpenMutationMiner (OMM), MutationFinder (MF), tmVar and SNP Extraction Tool for Human Variations (SETH).

COSMIC	EMU	OMM	MF	SETH	tmVar
EMU OMM MF SETH tmVar	- 0.1830 0.0266 0.3315 0.1682	0.7939 - 0.1623 0.7840 0.4325	0.5114 0.9783 - 0.0498 0.5056	0.7546 0.6065 0.0064 - 0.3423	0.6962 0.6987 0.1334 0.7077 -

**Table 13.** Overlap of mutations extracted by each tool on the InSiGHT database from all article data sources. The tools are Extractor of Mutations (EMU), OpenMutationMiner (OMM), MutationFinder (MF), tmVar and SNP Extraction Tool for Human Variations (SETH).

InSiGHT	EMU	OMM	MF	SETH	tmVar
EMU OMM MF SETH tmVar	- 0.1797 0.1459 0.0712 0.5162	0.1463 - 0.7134 0.0488 0.0976	0.1367 1.0000 - 0.0000 0.0171	0.3300 0.2700 0.0000 - 0.2700	0.7249 0.2169 0.0106 0.1429 -

Intrinsic results show that Open Mutation Miner and SETH have the best performance for protein and DNA mutations respectively. Table 14 shows that the combination recovers a large proportion of the mutations for the COSMIC database. On the other hand, the tools still fail to identify some genetic variants, mainly when they do not follow the HGVS format, as is the case in the supplementary material in InSiGHT. When we add EMU, as shown in Table 15, the coverage increases for InSiGHT, being close to the coverage obtained by combining the different tools.

**Table 14.** COSMIC and InSiGHT variant extraction coverage combining OMM and SETH. The data sets considered are MEDLINE abstracts (medline), Open Access PMC articles (pmc.ft), PDF articles when no Open Access PMC articles are available (pdf), tables available from the Open Access PMC Articles’ XML (table), supplementary material (sup) and the combination from all the sources (all).

Database	Data set	PMIDS	Total	Matched	Recall
COSMIC COSMIC COSMIC COSMIC COSMIC	medline pmc.ft pdf table sup	129 341 11 182 95	33814 33814 33814 33814 33814	145 736 7 640 22562	0.0043 0.0218 0.0002 0.0189 0.6672
COSMIC	all	400	33814	23544	0.6963
**Database**	**Data set**	**PMIDS**	**Total**	**Matched**	**Recall**
InSiGHT InSiGHT InSiGHT InSiGHT InSiGHT	medline pmc.ft pdf table sup	3 6 2 4 1	252 252 252 252 252	5 8 7 48 0	0.0198 0.0317 0.0278 0.1905 0.0000
InSiGHT	all	9	252	61	0.2421

**Table 15.** COSMIC and InSiGHT variant extraction coverage combining OMM, SETH and EMU. The data sets considered are MEDLINE abstracts (medline), Open Access PMC articles (pmc.ft), PDF articles when no Open Access PMC articles are available (pdf), tables available from the Open Access PMC Articles’ XML (table), supplementary material (sup) and the combination from all the sources (all).

Database	Data set	PMIDS	Total	Matched	Recall
COSMIC COSMIC COSMIC COSMIC COSMIC	medline pmc.ft pdf table sup	149 380 68 222 112	33814 33814 33814 33814 33814	151 794 34 692 22752	0.0045 0.0235 0.0010 0.0205 0.6729
COSMIC	all	511	33814	23826	0.7046
**Database**	**Data set**	**PMIDS**	**Total**	**Matched**	**Recall**
InSiGHT InSiGHT InSiGHT InSiGHT InSiGHT	medline pmc.ft pdf table sup	3 9 3 4 1	252 252 252 252 252	5 26 7 64 88	0.0198 0.1032 0.0278 0.2540 0.3492
InSiGHT	all	12	252	152	0.6032

## Discussion

The Results section presented results for two types of experiments. The first one compares the coverage of current mutation extraction tools on a data set intended to support the curation of the InSiGHT database. The second type of experiment looks at the performance of the tools in the context of mutation database curation.

The result on mutation extraction shows that performance is quite high using partial matching, with a result over 85 in F1 measure by SETH. We see a big change from the performance of the mutation extraction tools between exact and partial matching regimes, mainly due to the differences in boundaries due to annotation of the prefixes “c.” and “p.” in the gold standard, while not included by the extraction tools.

The split between DNA and protein variants shows the varying scope of the tools. OMM shows a high recall of protein mutations, only missing the mentions *M23A* and *V600E*, which were sourrounded by parentheses. MutationFinder missed, in addition to the OMM examples, protein variants using three letter amino acid abbreviations, e.g. *p.Pro622Thr*. In addition, MutationFinder was developed on citations available from PDB. It is quite likely that the mutations from these citations have been introduced by mutagenesis, instead of the natural gene variants covered in COSMIC and InSiGHT, and thus its performance might be influenced by this. EMU missed protein variants surrounded by parenthesis too, and mutations for which the amino acid substitution was specified by an X, e.g. *p.Arg226X*. SETH missed some mentions including variants without the *p.* protein variant prefix.

Considering the DNA variant annotation performed by EMU, tmVar and SETH, we find that all the tools missed expressions such as *codon 33: C>A*, expression that resembles the protein variants *A1796* and very complex expressions. For example, c. 423 -6delAAATAGGTinsGAAGCAAGATCAG in PMID:18433509. EMU missed DNA variants that seem more complex, with ranges in the substitution *c.1852_1853AA>GC* or other expressions *c.4236del8ins13*. tmVar missed some DNA mutations. Many were verbose expressions, e.g. *1799T to A*. SETH, in addition to the examples mentioned above, missed expressions that do not exactly follow the HGVS nomenclature, e.g. *C1668C > T*, even though it recovered the larger set of DNA variants.

Furthermore, the remaining mutation entities without change and location information include terms that are related to mutation. In some cases, there are terms that denote the location of the mutation but not the specific change, e.g. *exon 10*, mention the change without specifying the position, e.g. *G:C to A:T transition*, name a mutated gene *APC+*, and terms that describe the type of mutation *somatic mutation*. The variety of information covered by these terms might require the use of different techniques for each type. The first three types could be annotated using more general regular expressions, while a dictionary approach might be suitable for the terms describing the mutation types.

We processed the mutation types with the NCBO annotator ^38^ ( http://bioportal.bioontology.org/annotator), which uses a large set of terminologies and ontologies including the NCI thesaurus ^39^ and the Sequence Ontology ^40^. Some of the terms are properly annotated with concepts from these resources, e.g. *somatic mutation* or *germline mutation* while others are not covered by any of these resources, e.g. *truncating mutation*, including resources such as the Unified Medical Language System (UMLS) ^41^. Some terms are partially annotated but some simple rules could be considered to fully annotate them, for example, *pathogenic mutation* where *pathogenic* and *mutation* are annotated with different concepts or *large mutation* where only *mutation* is annotated.

Results on the recovery of curated mutations s how two things. First, recall of curated mutations from the narrative part of the documents is very low. Second, a large number of them can be recovered from tables and supplementary material. These results echo our previous results obtained using the EMU system alone ^11, 12^, and demonstrate that the complete set of material associated with a publication is commonly considered in curation of mutation information.

There are mutations not extracted by EMU that have been found by other mutation extraction methods. Most of the annotations missed by EMU are deletions and duplicates that seem to be partly covered by EMU but are better covered by tools like SETH. There are many protein mutations missed by SETH, because of the lack of an explicit *p.* prefix, that are covered by Open Mutation Miner.

The most significant increase in recall of the COSMIC database happens in the supplementary material. The recall of the combination increases from 0.5671 to 0.6730. The overall recall, which was around 0.52 for EMU alone, increases to 0.7053 for the combined outputs.

In the InSiGHT database, the most significant increase in recall takes place when adding the mutations extracted from the tables. The main reason for this is the SETH tool’s identification of deletions in the tables. The overall recall, which was previously around 0.45 using EMU, increases to 0.6230 when combining the output of all the mutation tools.

We have annotated the variants available in each of the databases with a mutation type, using SETH. SETH, as mentioned in the methods section, annotates mutations based on grammar defined for HGVS ^23^ and produces a mutation type for each string it recognizes. The list of mutation types is: SUBSTITUTION, DELETION, DELETION_INSERTION, DUPLICATION, INSERTION, FRAMESHIFT. To this set, we have added the type UNKNOWN in the case SETH does not identify a specific mutation type, typically caused by underspecified phrases such as *c.?* and *p.?*. For a few cases like the substitution *p.H776_C777>QS* SETH also does not return any mutation type. There are many cases in which a DNA mutation cannot be mapped to a protein mutation. Frameshift mutations like *L280FfsX4* are not covered by SETH. On the other hand, neither *p.H776_C777>QS* or *L280FfsX4* follow the HGVS nomenclature. This implies that there is a small portion of the mutations coded in the mutation databases that are not fully compliant with the HGVS nomenclature.

The mutations in the InSiGHT and COSMIC databases are already in HGVS format, and so SETH can be used directly to classify each mutation in the databases by variant type. The analysis by type is available in Table 16 and Table 17. We can see that a large proportion of the variants are substitutions. The analysis of missed variants by type is available in Table 18 and Table 19.

**Table 16.** COSMIC database variants, taking into account the DNA variant and the impact on the gene product. Variants have been annotated using SETH, thus the variant types delivered by SETH are considered.

Frequency	DNA variant type	Protein variant type
30080 1051 1012 490 351 215 179 171 63 59 53 38 26 19 3 3 1	SUBSTITUTION SUBSTITUTION DELETION UNKNOWN INSERTION DELETION UNKNOWN DELETION INSERTION UNKNOWN UNKNOWN INSERTION UNKNOWN SUBSTITUTION DELETION SUBSTITUTION DUPLICATION	SUBSTITUTION UNKNOWN FRAMESHIFT SUBSTITUTION FRAMESHIFT DELETION UNKNOWN UNKNOWN INSERTION DELETION FRAMESHIFT UNKNOWN INSERTION FRAMESHIFT SUBSTITUTION INSERTION UNKNOWN

**Table 17.** InSiGHT database variants, taking into account the DNA variant and the impact on the gene product. Variants have been annotated using SETH, thus the variant types delivered by SETH are considered.

Frequency	DNA variant type	Protein variant type
134 25 22 16 12 12 8 7 4 2 2 2 2 1 1 1 1	SUBSTITUTION SUBSTITUTION DELETION DELETION DELETION DUPLICATION UNKNOWN DELETION DUPLICATION DELETION_INSERTION DUPLICATION SUBSTITUTION SUBSTITUTION DUPLICATION DUPLICATION INSERTION UNKNOWN	SUBSTITUTION UNKNOWN FRAMESHIFT DELETION UNKNOWN FRAMESHIFT SUBSTITUTION SUBSTITUTION SUBSTITUTION SUBSTITUTION UNKNOWN DELETION FRAMESHIFT DUPLICATION INSERTION FRAMESHIFT DELETION

**Table 18.** COSMIC missing variants by the combination of variant extraction tools grouped by type, taking into account the DNA variant and the impact on the gene product. Missing variants have been annotated using SETH, thus the variant types delivered by SETH are considered.

Frequency	DNA variant type	Protein variant type
7163 805 759 276 191 171 155 60 56 43 36 33 25 19 3 3 1	SUBSTITUTION SUBSTITUTION DELETION INSERTION DELETION UNKNOWN DELETION UNKNOWN INSERTION UNKNOWN INSERTION UNKNOWN UNKNOWN SUBSTITUTION SUBSTITUTION DELETION DUPLICATION	SUBSTITUTION UNKNOWN FRAMESHIFT FRAMESHIFT DELETION UNKNOWN UNKNOWN SUBSTITUTION INSERTION DELETION UNKNOWN FRAMESHIFT INSERTION FRAMESHIFT INSERTION SUBSTITUTION UNKNOWN

**Table 19.** InSiGHT missing variants by the combination of variant extraction tools grouped by type, taking into account the DNA variant and the impact on the gene product. Missing variants have been annotated using SETH, thus the variant types delivered by SETH are considered.

Frequency	DNA variant type	Protein variant type
18 17 12 11 9 8 8 3 2 2 1 1 1 1 1	DELETION SUBSTITUTION DELETION DUPLICATION DELETION UNKNOWN SUBSTITUTION DELETION SUBSTITUTION DUPLICATION UNKNOWN SUBSTITUTION INSERTION DUPLICATION DUPLICATION	FRAMESHIFT UNKNOWN DELETION FRAMESHIFT UNKNOWN SUBSTITUTION SUBSTITUTION SUBSTITUTION DELETION UNKNOWN DELETION FRAMESHIFT FRAMESHIFT SUBSTITUTION INSERTION

In COSMIC, the most common type of missed mutations are DNA substitutions, mostly from two papers. These articles are PMID:21720365 with 5,589 variants and PMID:18772890 with 1,112 variants. The variants appear spread within supplementary material and in tables. We had already observed this previously ^11^, although here we perform more detailed annotation of the entities. Most of the DNA substitutions result in a known protein mutation, although there are around 805 mutations for which the effect on the protein is unknown.

In contrast to the large number of substitutions missing from the COSMIC database, DNA deletions are the most common variant type missing in the InSiGHT database. In some cases this is due to the failure of the text mining approaches. For instance, in PMID:15655560, only the substring as *1705delAG* of the deletion *c.1704_1705delAG* is identified by the combination of text mining tools. In addition, many of the deletions are not in a form usually expected by the mutation tools. This is the case of deletions expressed in non-standard nomenclature, e.g. *del exon 3*, as well as substitutions or deletions that require transformations, such as *IVS17(+5)G>C*, found in a table in PMID:14970868. The mutation extraction tools would need to take a closer look at these examples and incorporate the appropriate patterns.

There are mutations that are extracted by the mutation tools that do not appear in the curated databases, as found by Schench *et al.* ^30^. This is because these mutations are not of the interest to the databases. This also explains the low number of matches between InSiGHT and COSMIC within the 40 overlapping articles. COSMIC focuses on somatic mutations while InSiGHT collects germline mutations related to Lynch Syndrome for just four genes. In addition, some of the extracted mentions are not functional or significant for the disease, as previously described ^11^. For instance in PMID:10469011, the mutation Ala140Thr is extracted but the article states this mutation … *is known to be functionally silent*, and hence was excluded from the database.

We have looked at the types of mutations annotated by each tool, using SETH to identify the mutation type. These are available from Table 20 and Table 21. Similarly to existing results, most of the mutations are substitutions and in lower number, deletions and duplications. The tools do not reliably identify insertions, although a large number of variants that could not be annotated by SETH, and were labelled as UNKNOWN, are actually insertions. A list of these mutations is available from the bitbucket repository https://bitbucket.org/readbiomed/mutationtoolcomparison. As expected, OMM and MF do not annotate DNA variants and annotate protein substitutions.

**Table 20.** DNA variants annotated by each mutation extraction tool, using all the sources from the articles, tool grouped by type. The type of each variant has been annotated using SETH, thus the variant types delivered by SETH are considered. The tools are Extractor of Mutations (EMU), OpenMutationMiner (OMM), MutationFinder (MF), tmVar and SNP Extraction Tool for Human Variations (SETH). A large number of DNA mutations are annotated by EMU. 1887448 of the COSMIC variants are substitutions derived from the supplementary material of a single article, PMID:22622578. Most of these substitutions are provided in terms of chromosome location rather than relative to the gene.

Tool	Type	Frequency (COSMIC)	Frequency (InSiGHT)
EMU	All SUBSTITUTION DELETION UNKNOWN INSERTION DELETION_INSERTION	1914828 1914100 405 186 133 4	182 172 - 7 3 -
OMM	-	-	-
MF	-	-	-
SETH	All SUBSTITUTION DELETION UNKNOWN DUPLICATION	9029 8212 673 113 31	58 25 22 0 11
tmVar	All SUBSTITUTION DELETION UNKNOWN DUPLICATION	1864 1065 464 313 22	135 113 12 10 0

**Table 21.** Protein variants annotated by each mutation extraction, using all the sources from the articles, tool grouped by type. The type of each variant has been annotated using SETH, thus the variant types delivered by SETH are considered. The tools are Extractor of Mutations (EMU), OpenMutationMiner (OMM), MutationFinder (MF), tmVar and SNP Extraction Tool for Human Variations (SETH).

Tool	Type	Frequency (COSMIC)	Frequency (InSiGHT)
EMU	All SUBSTITUTION SILENT UNKNOWN	35871 35560 157 154	102 100 2 -
OMM	SUBSTITUTION	29157	164
MF	SUBSTITUTION	4836	117
SETH	All SUBSTITUTION UNKNOWN	28862 28753 109	42 41 1
tmVar	All SUBSTITUTION DELETION UNKNOWN	16461 16352 90 19	54 53 1 -

Supplementary material for the COSMIC database and tables for the InSiGHT database contribute a large number of mutations. On the other hand, processing these type of resources poses new challenges. Examination of the supplementary files show that many of the mutations listed in MS Excel files and MS Word files are in a tabular format, thus the problem could be reduced to mutation extraction from tables. Table processing for mutation extraction has been already explored by Wong *et al.* ^42^. The scope of that work was to classify the type of information in each field in a table, based on a machine-learned model. This work could be extended to properly identify the gene associated to a mutation, even if it appears in the caption of the table, in a column header, and/or as a field in the same row as the mutation. This requires additional mechanisms to extract additional fields and post-process the extracted information in order to normalize the mutation mentions.

## Conclusions

We have performed a broad assessment of the state-of-the-art performance of existing tools for extraction of genetic variants from the published biomedical literature, considering and comparing five publicly available extraction tools on two complementary evaluation tasks. We have proposed combining multiple mutation extraction tools together by merging their results, and have shown that their combination results in substantially improved recall of mutations with minimal impact on precision, providing evidence of the complementary nature of the tools.

Our results show that current tools have a very good performance on the narrative parts of published articles, and demonstrate that earlier performance claims for MEDLINE abstracts extend to full text. On the other hand, the excellent extraction performance on this narrative content contrasts with substantially lower recall in the context of database curation, even when all results of all considered tools are merged together. Our results demonstrate that only a small fraction of the curated mutations are available from the narrative part of the articles, and that most of the information is available only in tables and supplementary files associated to the articles. When the tools are deployed against this additional material, we are able to substantially increase recall. This increase is particularly evident when the tools are used together.

We have further examined in detail the performance of these tools on different types of genetic variants, considering not only the distinction between protein variation and DNA variation, but also contrasting performance on different types of variation, e.g. substitutions, deletions, and insertions. We demonstrate that the coverage of different tools is quite complementary with respect to these distinctions, providing an explanation for the performance benefit obtained by merging their results.

Future work involves integrating our results into a genetic variant database curation tool. Before achieving this goal, there are several improvements to perform. As we have seen, the combination of mutation extraction tools recover a large part of the mutations curated in existing databases, and therefore combining several tools together is a viable strategy for genetic variant extraction, but there are still variants that are not covered. Our error analysis shows that a particular gap is mutations that do not include an explicit location or that imply a variation in a specific region in a gene (e.g. *Del exon 3*). Coverage of DNA insertions is low, and could be a particular target for improvement.

Furthermore, special processing is required to recover information from tables. To address this, we plan to extend work previously done by Wong *et al.* ^42^. Finally, a curation tool must consider the differing scopes of different genetic variant databases, i.e. COSMIC is interested in somatic mutations while InSiGHT is interested in germline mutations. Further extension to this work would therefore include classifying the variants into these two categories.

## References

[1] Hamosh A Scott AF Amberger JS: Online Mendelian Inheritance in Man (OMIM), a knowledgebase of human genes and genetic disorders. *Nucleic Acids Res.* 2005;33(Database issue):D514–D517 10.1093/nar/gki033 15608251 PMC539987

[2] Claustres M Horaitis O Vanevski M: Time for a unified system of mutation description and reporting: A review of locus-specific mutation databases. *Genome Res.* 2002;12(5):680–688 10.1101/gr.217702 11997335

[3] Baker CJO René W: Mutation Mining: A Prospector’s Tale. *Journal of Information Systems Frontiers.* 2006;8(1):47–57 10.1007/s10796-006-6103-2

[4] Caporaso JG Baumgartner WA Randolph DA: MutationFinder: A high-performance system for extracting point mutation mentions from text. *Bioinformatics.* 2007;23(14):1862–1865 10.1093/bioinformatics/btm235 17495998 PMC2516306

[5] Krallinger M Izarzugaza JM Rodriguez-Penagos C: Extraction of human kinase mutations from literature, databases and genotyping studies. *BMC Bioinformatics.* 2009;10(Suppl 8):S1 10.1186/1471-2105-10-S8-S1 19758464 PMC2745582

[6] Doughty E Kertesz-Farkas A Bodenreider O: Toward an automatic method for extracting cancer- and other disease-related point mutations from the biomedical literature. *Bioinformatics.* 2011;27(3):408–415 10.1093/bioinformatics/btq667 21138947 PMC3031038

[7] Naderi N Witte R: Automated extraction and semantic analysis of mutation impacts from the biomedical literature. *BMC Genomics.* 2012;13(Suppl 4):S10 10.1186/1471-2164-13-S4-S10 22759648 PMC3395893

[8] Hakenberg J Voronov D Nguyên VH: A SNPshot of PubMed to associate genetic variants with drugs, diseases, and adverse reactions. *J Biomed Inform.* 2012;45(5):842–50 10.1016/j.jbi.2012.04.006 22564364

[9] Wei CH Harris BR Kao HY: tmVar: a text mining approach for extracting sequence variants in biomedical literature. *Bioinformatics.* 2013;29(11):1433–1439 10.1093/bioinformatics/btt156 23564842 PMC3661051

[10] den Dunnen JT Antonarakis SE: Mutation nomenclature extensions and suggestions to describe complex mutations: a discussion. *Hum Mutat.* 2000;15(1):7–12 10.1002/(SICI)1098-1004(200001)15:1<7::AID-HUMU4>3.0.CO;2-N 10612815

[11] Jimeno Yepes A Verspoor K: Literature mining of genetic variants for curation: Quantifying the importance of supplementary material. *Database: The Journal of Biological Databases and Curation.* 2014;2014:bau003 10.1093/database/bau003 24520105 PMC3920087

[12] Jimeno-Yepes A Verspoor K: Towards automatic large-scale curation of genomic variation: improving coverage based on supplementary material.In *Proceedings of BioLINK SIG 2013* 2013;39–43 Reference Source

[13] Bamford S Dawson E Forbes S: The COSMIC (Catalogue of Somatic Mutations in Cancer) database and website. *Br J Cancer.* 2004;91(2):355–358 10.1038/sj.bjc.6601894 15188009 PMC2409828

[14] Plazzer JP Sijmons RH Woods MO: The InSiGHT database: Utilizing 100 years of insights into Lynch Syndrome. *Familial Cancer.* 2013;12(2):175–180 10.1007/s10689-013-9616-0 23443670

[15] Verspoor K Jimeno Yepes A Cavedon L: Annotating the biomedical literature for the human variome. *Database (Oxford)* 2013;2013:bat019 10.1093/database/bat019 23584833 PMC3676157

[16] Xuan W Wang P Watson SJ: Medline search engine for finding genetic markers with biological significance. *Bioinformatics.* 2007;23(18):2477–2484 10.1093/bioinformatics/btm375 17823133

[17] Thomas P Rocktäschel T Mayer Y: SETH: SNP extraction tool for human variations.2014 Reference Source

[18] Nagel K Jimeno-Yepes A Rebholz-Schuhmann D: Annotation of protein residues based on a literature analysis: Cross-validation against UniProtKb. *BMC Bioinformatics.* 2009;10(Suppl 8):S4 10.1186/1471-2105-10-S8-S4 19758468 PMC2745586

[19] Nagel K: Automatic functional annotation of predicted active sites: Combining PDB and literature mining. PhD thesis, University of Cambridge.2009 Reference Source

[20] Ravikumar K Liu H Cohn JD: Literature mining of protein-residue associations with graph rules learned through distant supervision. *J Biomed Semantics.* 2012;3(Suppl 3):S2 23046792 10.1186/2041-1480-3-S3-S2 PMC3465209

[21] Sherry ST Ward MH Kholodov M: dbSNP: the NCBI database of genetic variation. *Nucleic Acids Res.* 2001;29(1):308–311 10.1093/nar/29.1.308 11125122 PMC29783

[22] Comeau DC Islamaj Doğan R Ciccarese P: BioC: a minimalist approach to interoperability for biomedical text processing. *Database: The Journal of Biological Databases and Curation.* 2013;2013:bat064 10.1093/database/bat064 24048470 PMC3889917

[23] Jeroen JF Blavier A den Dunnen JT: A formalized description of the standard human variant nomenclature in Extended BackusNaur Form. *BMC Bioinformatics.* 2011;12(Suppl 4):S5 10.1186/1471-2105-12-S4-S5 21992071 PMC3194197

[24] Thomas PE Klinger R Furlong L: Challenges in the association of human single nucleotide polymorphism mentions with unique database identifiers. *BMC Bioinformatics.* 2011;12(Suppl 4):S4 10.1186/1471-2105-12-S4-S4 21992066 PMC3194196

[25] Furlong LI Dach H Hofmann-Apitius M: OSIRISv1. 2: a named entity recognition system for sequence variants of genes in biomedical literature. *BMC Bioinformatics.* 2008;9(1):84 10.1186/1471-2105-9-84 18251998 PMC2277400

[26] Ortutay C Väliaho J Stenberg K: KinMutBase: a registry of disease-causing mutations in protein kinase domains. *Hum Mutat.* 2005;25(5):435–442 10.1002/humu.20166 15832311

[27] Yip YL Scheib H Diemand AV: The Swiss-Prot variant page and the ModSNP database: A resource for sequence and structure information on human protein variants. *Hum Mutat.* 2004;23(5):464–470 10.1002/humu.20021 15108278

[28] Hurst JM McMillan LE Porter CT: The SAAPdb web resource: A large-scale structural analysis of mutant proteins. *Hum Mutat.* 2009;30(4):616–624 10.1002/humu.20898 19191322

[29] Jia M Forbes SA Beare D: Mining cancer genomes in COSMIC. *In BMC Proceedings.* 2012;6(Suppl 6):17 10.1186/1753-6561-6-S6-P17

[30] Schenck M Politz O Groth P: Extraction of genetic mutations associated with cancer from public literature. *J Health Med Informat.* 2012; (S2). 10.4172/2157-7420.S2-002

[31] Caporaso JG Deshpande N Fink JL: Intrinsic evaluation of text mining tools may not predict performance on realistic tasks. *Pac Symp Biocomput.* 2008;640–651 18229722 PMC2517250

[32] Verspoor K MacKinlay A Cohn JD: Detection of protein catalytic sites in the biomedical literature. *Pac Symp Biocomput.* 2013;18:433–444 10.1142/9789814447973_0042 23424147 PMC3664919

[33] Rance B Doughty E Demner-Fushman D: A mutation-centric approach to identifying pharmacogenomic relations in text. *J Biomed Inform.* 2012;45(5):835–841 10.1016/j.jbi.2012.05.003 22683993 PMC3438287

[34] Verspoor K Cohen KB Hunter L: The textual characteristics of traditional and Open Access scientific journals are similar. *BMC Bioinformatics.* 2009;10:183 10.1186/1471-2105-10-183 19527520 PMC2714574

[35] Jimeno-Yepes JA Sticco JC Mork JG: GeneRIF indexing: sentence selection based on machine learning. *BMC Bioinformatics.* 2013;14:171 10.1186/1471-2105-14-171 23725347 PMC3687823

[36] MacKinlay A Verspoor K: A Web Service Annotation Framework for CTD Using the UIMA Concept Mapper. *BioCreative Challenge Evaluation Workshop.* 2013;1 Reference Source

[37] Michael AT Anni C Igor LS: The ConceptMapper Approach to Named Entity Recognition. *LREC.* 2010 Reference Source

[38] Clement J Nigam S Cherie Y: NCBO annotator: semantic annotation of biomedical data. In *International Semantic Web Conference, Poster and Demo session* 2009 Reference Source

[39] Sioutos N de Coronado S Haber MW: NCI Thesaurus: a semantic model integrating cancer-related clinical and molecular information. *J Biomed Inform.* 2007;40(1):30–43 10.1016/j.jbi.2006.02.013 16697710

[40] Eilbeck K Lewis SE Mungall CJ: The Sequence Ontology: a tool for the unification of genome annotations. *Genome Biol.* 2005;6(5):R44 10.1186/gb-2005-6-5-r44 15892872 PMC1175956

[41] Bodenreider O: The Unified Medical Language System (UMLS): integrating biomedical terminology. *Nucleic Acids Res.* 2004;32(suppl 1):D267–D270 10.1093/nar/gkh061 14681409 PMC308795

[42] Wong W Martinez D Cavedon L: Extraction of named entities from tables in gene mutation literature. *BioNLP.* 2009;46–54 Reference Source

