# Supplementary material for: Mutation extraction tools can be combined for robust recognition of genetic variants in the literature
Source: F1000Res. 2014 Jun 10;3:18. Originally published 2014 Jan 21. [Version 2] doi: 10.12688/f1000research.3-18.v2 (PMC4176422; doi:10.12688/f1000research.3-18.v2)
Supplement: Peer review history

# Peer review: Referee response for version 2

**DOI:** 10.5256/f1000research.4577.r5372

**Published:** 2014-07-08

**Authors:** Cecilia N Arighi

**Affiliations:** 1 Center for Bioinformatics and Computational Biology, University of Delaware, Newark, DE, USA

The authors have responded satisfactorily to my comments and explained where some items could not be addressed.

I have no further comments.

I have read this submission. I believe that I have an appropriate level of expertise to confirm that it is of an acceptable scientific standard.

# Peer review: Referee response for version 1

**DOI:** 10.5256/f1000research.3422.r3722

**Published:** 2014-03-28

**Authors:** Cecilia N Arighi

**Affiliations:** 1 Center for Bioinformatics and Computational Biology, University of Delaware, Newark, DE, USA

This article shows a thorough evaluation of the performance of five text mining tools for mutation extraction. The authors perform two types of evaluation: one to test the ability of the tools in identifying specific mentions of genetic variation in the manually-annotated Variome corpus and the other, in the context of database curation, to compare results against the data in COSMIC and InSiGHT databases. The authors analyzed the tools both individually and combined.

The main findings include:

- The tools have complementary coverage of genetic variants mentioned in the literature (e.g. some tools covered only single point mutations whereas others covered deletions and insertions as well), therefore the combination of tools proves to be more effective.
- The external evaluation on the database corpora is important to assess the utility of the tools for biocuration.
- The main results derive from the location of the data in the article.
- The majority is located in the Supplementary materials (for COSMIC) and also in Tables (for InSiGHT).
- The authors also demonstrated that the tools, even when they were developed for medline abstracts, can be applied to other text.  

The article is well written, provides sufficient background information, is scientifically sound, and both the title and abstract reflect the main research results and conclusions of this work. However, there is still room for improvement.

Here are some suggestions:

1. The article talks about mutations referring more narrowly to natural gene variants (at least that would be the case for the databases mentioned above). However, at least one tool (MutationFinder) seems to consider mutation in a broader sense. The fact that the corpus was based on PMIDs in PDB suggests that these are most likely to be mutations introduced by mutagenesis (e.g. I checked a couple of examples of the corpus used by MF and, in fact, some are mutagenesis experiments) rather than genetic variants. This may also affect the performance of the tool if most of the examples that it was exposed to were of these kind. I would suggest including some comment on this in the article.
2. The description of TmVar in the background section should include the type of variant it is able to extract. This description is present in all other systems and should be described in the paragraph where they are referred to in the paper.
3. Both exact and partial matches are used for the calculation of performance. Partial match is simply defined as “ *annotated entities may have any overlap with the entities in the reference set*”. Have you analyzed the range of percent overlap?
4. Tables 8, 9, 14 and 15 need some clarification. I understand that the point the authors want to convey is about the coverage not only of the tools, but of the data in the various sections of the article (main text, tables, supplementary material, abstract vs full length).The value therefore, that is presented in the “Recall” column is like a fractional recall, as it is compared to the total number of variants in the reference set. The last row is probably the only line presenting the total Recall measure. The table is missing a row before the last one with Dataset:ALL, Tool: each tool. This row and the previous one should be highlighted as these are the ones showing the total recall.
5. I would also add a column with the number of articles and annotations in each of the data sets (medline, pmc, pdf, etc), and the performance at each of these levels, i.e. calculate the recall on a section (Medline) based on the total annotation present in such section.  
6. I would change the title of Recall for the one showing fractional recall (calculated against Total). Maybe use a term such as coverage fraction or coverage recall? (Except for the Data set all).

I have read this submission. I believe that I have an appropriate level of expertise to confirm that it is of an acceptable scientific standard, however I have significant reservations, as outlined above.

# Response

**Published:** 2014-05-20

**Authors:** Antonio José Jimeno Yepes

**Affiliations:** NICTA, Australia

Thank you for your suggestions. You can find our answers to the points you raised below:

1. This is an important detail that allows for a better understanding of the behaviour by MutationFinder. We have added the following sentence in the discussion section: *In addition, MutationFinder was developed on citations available from PDB. It is quite likely that the mutations from these citations have been introduced by mutagenesis, instead of the natural gene variants covered in COSMIC and InSiGHT, and thus its performance might be influenced by this.*

2. Thank you for pointing this out. The following sentence has been added to the manuscript: *tmVar aims to cover a wide range of sequence variants in both protein and gene levels in HGVS format.*

3. This is a good idea since it provides a better overview of the matched mutations. We have added the information about the character overlap in tables 3, 4 and 5. Discussion about the results has been added to the manuscript.

4. A set of rows for *Dataset:All* have been added in tables 8 to 10. 

5. This would be an interesting result. Unfortunately, the curated databases used for this extrinsic evaluation do not offer this level of detail, i.e. it is not possible to tell from which source the information was curated from (i.e. whether the curator used an abstract, full text, etc.).

6. Since the recall per data set section cannot be obtained, we have decided to leave the current recall label.

# Peer review: Referee response for version 1

**DOI:** 10.5256/f1000research.3422.r3961

**Published:** 2014-03-27

**Authors:** Max Haeussler

**Affiliations:** 1 Department of Biomolecular Engineering, University of California Santa Cruz, Santa Cruz, CA, USA

The authors have done the formidable job of running five programs that search for mutations in English text on 10 annotated full text articles (they call this "intrinsic") and also on articles that have been annotated by curators of two databases ("extrinsic"). The results make sense, they analyze them well, the article is very readable and the methods are suitable. The conclusions are relevant for everyone who is developing mutation mining tools, probably most users of text mining algorithms, and they are well justified.

Why I have reservations: The authors were able to conduct this study thanks to a great practice in bioinformatics, namely to provide the source code of the program used in a scientific article. They were able to download the source code of at least three programs from other websites or supplemental data and were even able to benchmark a program which has not been published yet in an article (SETH), and found that it led the field. This shows how the state of the art progresses by sharing code (even without publications) - the authors did not have to write their own mutation finding tool, they could run existing programs on a new corpus.

However, it is hard to understand why the authors do not do the same. Anyone who would like to inspect or extend the results of this article would have to redo the same work again: write parsers for five programs, convert both corpora, put the main corpus into a suitable format, including downloading some files from EuropePMC, others from PMC, and write a program to evaluate the differences.

I fully agree with Referee 1 ( **Philippe Thomas**) that when claims about performance of open source programs are made that have been written by various teams, then at least the corpus + annotations have to be added as a supplemental file. Otherwise it is very hard for a field to improve their algorithms and validate claims made in the benchmark.

In this case, the code for running the mutation detection programs should also be provided. Philippe Thomas **'** (the author of SETH) comment that the results of SETH should have been better for a particular case can only be answered by looking at the code, the version (or the github commit ID in case of pre-publication code) the authors used and the converter, to find out where the differences come from. For a reader, it is currently impossible to validate the observation about SETH. This could be really easy to change, if the authors just attached their corpus+program as a supplemental file or on github, like most other authors in this field today.

I have read this submission. I believe that I have an appropriate level of expertise to confirm that it is of an acceptable scientific standard, however I have significant reservations, as outlined above.

# Response

**Published:** 2014-05-20

**Authors:** Antonio José Jimeno Yepes

**Affiliations:** NICTA, Australia

Thank you for your suggestions. We have made available evaluation files in a bitbucket repository ( https://bitbucket.org/readbiomed/mutationtoolcomparison). The brat mutation annotation files for the Variome corpus evaluation have been included as well. In addition, the tool brateval ( https://bitbucket.org/nicta_biomed/brateval) can be used to reproduce the evaluation presented in the paper for the experiments with the Variome corpus. There are three sets of annotation files. One for all the mutations and then one set for the protein mutations only and another one for the DNA mutations only.

The benchmark files for the database evaluation have been made available for COSMIC and for InSiGHT. We have made available the PMIDs of the articles and the genes and mutations curated in each one of them.

We have not redistributed the text of the articles since due to license restrictions it is not possible to redistribute them. They can be obtained from the providers of the documents as indicated in the paper. The code to collect the data from PMC has been provided.

# Peer review: Referee response for version 1

**DOI:** 10.5256/f1000research.3422.r3233

**Published:** 2014-02-05

**Authors:** Philippe Thomas

**Affiliations:** 1 Knowledge Management in Bioinformatics, Computer Science Department, Humboldt-Universität zu Berlin, Berlin, Germany

This article comprehensively evaluates a series of named entity recognition tools for identifying mutation mentions. The different tools are evaluated using two kinds of scenarios: First, they are evaluated in an intrinsic setting, using the manually annotated "Variome" corpus. This strategy represents the typical evaluation of named entity recognition tools. Second, tools are compared in an extrinsic context, where the goal is to reconstruct mutational information contained in the COSMIC and InSiGHT databases. In the latter experiment, the authors identified supplemental material as the biggest resource for mutation mentions. The overall verdict of this publication is that several tools have complementary coverage and combination of methods leads to a performance increase in both evaluation settings. Finally, the authors provide some ideas to improve the recognition of genetic variants in text.

This work is scientifically sound and provides an interesting analysis of five different mutation recognition tools. I only have a few remarks considering the presentation:

 

1. The results for the intrinsic evaluation, shown in Table 5, require some more details. For all tools a considerable increase of recall can be observed when switching from the exact matching to the partial matching scenario. However, for OpenMutationMiner, recall increases by almost 86 percentage points whereas the increase for the other tools is about 10 percentage points. This is a surprising result and should be at least briefly mentioned in the text.
2.  A frequently found statement is that SETH recognizes only mutation mentions described in HGVS nomenclature. However, as a participant in the SETH project (see competing interests), I suggest that this claim is incorrect as SETH builds on MutationFinder to detect mutations not following the nomenclature. Furthermore, SETH modifies MutationFinder's original capabilities in order to match a wider scope of mutations (DNA mutations, nonsense mutations, and ambiguous mutations) not following the HGVS nomenclature. This is done by modifying the original MutationFinder implementation together with additional and modified regular expressions. Furthermore, SETH contains a separate component (OldNomenclature.java) for the recognition of insertions, deletions, and frameshift mutations written in deprecated HGVS nomenclature. For these reasons it is surprising, that SETH achieves lower recall than MutationFinder in many extrinsic evaluation settings (Table 8 and Table 9). In my opinion, SETH should at least find as many mutations as MutationFinder.
3.  Please use the citation provided on the SETH project site to cite SETH. (Thomas, P., Rocktäschel, T., Mayer, Y., and Leser, U. (2014). SETH: SNP Extraction Tool for Human Variations. http://rockt.github.io/SETH/. )

I also have a list of minor remarks, which I would like to mention here. However, these are just ideas to further improve the publication.

- Table 3 shows that merging results of all tools increases performance in the partial evaluation setting. It would be interesting to see how this compares to exact matching. Do you see similar effects? It would be great to mention the numbers in the table.
- " *For a few cases like the substitution p.H776_C777>QS SETH also does not return any mutation type. There are many cases in which a DNA mutation cannot be mapped to a protein mutation and frameshift mutations like L280FfsX4 are not covered by SETH.*" I find this example a bit contrived, as neither p.H776_C777>QS nor L280FfsX4 follow the HGVS nomenclature.
- The sentence " *There are many cases in which a DNA mutation cannot be mapped to a protein mutation and frameshift mutations like L280FfsX4 are not covered by SETH*" on page 13 is (in my opinion) confusing and could be reformulated. If I understand correctly, you are saying that COSMIC and InSiGHT cannot provide protein mutations for all DNA mutations.
-  Integration of different tools usually is an ungrateful task. It would be very interesting if the authors could share some of their experiences made using the five different tools.
-  " *The tools do not reliably identify insertions, although a large number of variants that could not be annotated by SETH are actually insertions*." It would be highly interesting to see a list of these mutations (maybe as Supplemental data), to help tool developers improve named entity recognition tools. Additionally, I think that it would be very valuable to add all mutation mentions extracted by the five different tools to the Supplement as well. This information should facilitate the improvement of existing named entity recognition tools.

I have read this submission. I believe that I have an appropriate level of expertise to confirm that it is of an acceptable scientific standard, however I have significant reservations, as outlined above.

# Response

**Published:** 2014-05-20

**Authors:** Antonio José Jimeno Yepes

**Affiliations:** NICTA, Australia

Thank you for your comments and remarks. You can find the answers to the points you raised below:

1. Indeed, the increase in performance is significant. The reason is that some tools do not annotate the prefix of the mutation. For instance, in the corpus there is the mutation *p.Val600Glu* but OMM just annotates *Val600Glu.* This means that using exact match, all these mutations will be identified as false positives instead of true positives. We have added a statement to this effect in the text.

2. The claim about non HGVS compliance has been rewritten in the manuscript - The reason for this is that we have considered the version of SETH in which only its main components are used, which does not include MutationFinder. This point has been clarified in the paper.

3. This has been corrected in the manuscript.

The answers to your minor remarks can be found below:

1. We have updated the results in Table 3. There is an increase in recall in both cases and the results, which is a bit better than previously reported in the partial matching approach. We have corrected a problem with the tmVar input files and this explains further changes in the results.

2. Thank you for pointing this out. This implies as well that a small part of the mutations in the databases are not the fully compliant with the HGVS nomenclature. This point has been made in the manuscript with the following text: *On the other hand, neither p.H776_C777>QS or L280FfsX4 follow the HGVS nomenclature. This implies that there is a small portion of the mutations coded in the mutation databases are not fully compliant with the HGVS nomenclature.*

3. Thank you for pointing out this issue. The sentence has been rewritten as: *COSMIC and InSiGHT cannot provide protein mutations for all DNA mutations thus these mutations could not be annotated by SETH. Frameshift mutations like L280FfsX4 are not covered by SETH.*

4. Technical Notes have been added for each tool, in a brief “Technical Notes:” section for each tool.

5. A file has been added as supplementary material in a bitbucket repository ( https://bitbucket.org/readbiomed/mutationtoolcomparison), within the data folder, with these mutations and has been referenced in the paper.
